# Human mesenchymal stem cells lose their functional properties after paclitaxel treatment

**DOI:** 10.1038/s41598-017-18862-1

**Published:** 2018-01-10

**Authors:** Franziska Münz, Ramon Lopez Perez, Thuy Trinh, Sonevisay Sisombath, Klaus-Josef Weber, Patrick Wuchter, Jürgen Debus, Rainer Saffrich, Peter E. Huber, Nils H. Nicolay

**Affiliations:** 1 Heidelberg Institute for Radiation Oncology (HIRO), National Center for Radiation Research in Oncology, Neuenheimer Feld 280, 69120 Heidelberg, Germany; 2 Department of Molecular and Radiation Oncology, German Cancer Research Center (dkfz), Neuenheimer Feld 280, 69120 Heidelberg, Germany; 3 Department of Radiation Oncology, Heidelberg University Hospital, Neuenheimer Feld 400, 69120 Heidelberg, Germany; 4 Institute of Transfusion Medicine and Immunology, German Red Cross Blood Service Baden-Württemberg - Hessen, Medical Faculty Mannheim, Friedrich-Ebert-Str. 107, 68167 Mannheim, Germany; 5 Department of Hematology and Oncology, Heidelberg University Hospital, Neuenheimer Feld 410, 69120 Heidelberg, Germany

## Abstract

Mesenchymal stem cells (MSCs) are an integral part of the bone marrow niche and aid in the protection, regeneration and proliferation of hematopoietic stem cells after exposure to myelotoxic taxane anti-cancer agents, but the influence of taxane compounds on MSCs themselves remains incompletely understood. Here, we show that bone marrow-derived MSCs are highly sensitive even to low concentrations of the prototypical taxane compound paclitaxel. While MSCs remained metabolically viable, they were strongly impaired regarding both their proliferation and their functional capabilities after exposure to paclitaxel. Paclitaxel treatment resulted in reduced cell migration, delays in cellular adhesion and significant dose-dependent inhibition of the stem cells’ characteristic multi-lineage differentiation potential. Cellular morphology and expression of the defining surface markers remained largely unaltered. Paclitaxel only marginally increased apoptosis in MSCs, but strongly induced premature senescence in these stem cells, thereby explaining the preservation of the metabolic activity of functionally inactivated MSCs. The reported sensitivity of MSC function to paclitaxel treatment may help to explain the severe bone marrow toxicities commonly caused by taxane-based anti-cancer treatments.

## Introduction

The taxanes form a class of cytotoxic diterpene compounds that are widely used for the treatment of solid malignancies. The prototypical taxane drug paclitaxel as isolated from the bark of the Pacific yew tree was first described in the late 1960s to exhibit cytotoxic effects against tumor cells *in vitro* ^1^. Since then, the taxanes have shown beneficial effects in various clinical trials, and the compounds paclitaxel, docetaxel and cabazitaxel have been introduced into therapeutic regimens for ovarian, breast, bladder, prostate, esophageal and lung cancers as well as melanoma and Kaposi’s sarcoma^2–6^. Taxanes promote their cytotoxic effects by associating with GDP-bound tubulin proteins, thereby stabilizing polymerized microtobules and preventing the dynamic function of the cytoskeleton^7,8^; this in turn results in mitotic arrest, inhibition of cellular division and ultimately apoptotic cell death^9^.

Clinically, taxane-based treatment regimens commonly cause unfavourable and long-lasting side effects, including peripheral neuropathy, intestinal and bone marrow toxicities that often prove dose-limiting^10^. The observed toxicities are believed to be due to the sensitivity of tissue-specific stem cells to taxanes, and the duration of the observed side effects depends on the body’s intrinsic ability to regenerate, as to date no causative therapeutic approaches are available^11–13^.

Mesenchymal stem cells (MSCs) comprise a heterogeneous group of adult multipotent stromal cells that can be detected in many tissues, such as vascular and adipose tissues, bone marrow, kidney, skin and intestines^14,15^. The characterization of MSCs depends on a combination of functional and molecular criteria, including the cells’ ability to adhere to plastic surfaces, their expression of a distinct surface marker pattern and their potential for differentiation along the osteogenic, chondrogenic and adipogenic lineages^16,17^.

The regenerative abilities of MSCs have long been subject to intense research, and it has been shown that MSCs can participate in the repair of tissue lesions both by creating a regenerative microenvironment and by differentiating into functional, tissue-specific cells^18^. It has been demonstrated that stimulated endogenous MSCs can home into damaged tissues and exert their supportive effects locally^19,20^. MSCs have proved beneficial for the regeneration of organ damage caused by various anti-cancer agents such as cisplatin or bleomycin in animal models, and it is conceivable that these stem cells may also exert regenerative effects for similar toxicities observed after taxane-based treatments^21,22^. However, the influence of taxanes on MSCs themselves is widely unknown.

Here, we examined the effects of the prototypical taxane compound paclitaxel on the survival and proliferation of human bone marrow-derived MSCs. Potential effects of paclitaxel on the defining stem cell characteristics and functional capabilities were investigated and the influence on the induction of apoptosis and senescence was studied.

## Results

### Paclitaxel strongly reduces proliferation, but not viability of human MSCs

Taxane sensitivity of human MSCs and differentiated fibroblasts was assessed by metabolic viability and clonogenic survival assays. The paclitaxel treatment times and doses used for our experiments were based on the conditions reported for patients undergoing taxane chemotherapy: A previous metaanalysis analyzing 121 published pharmacokinetic profiles of taxane monotherapy had demonstrated an interstudy median maximum plasma concentration of 5100 nM and a drop in paclitaxel plasma concentration below 50 nM at 23.8 hours^23^.

**Figure 1:**
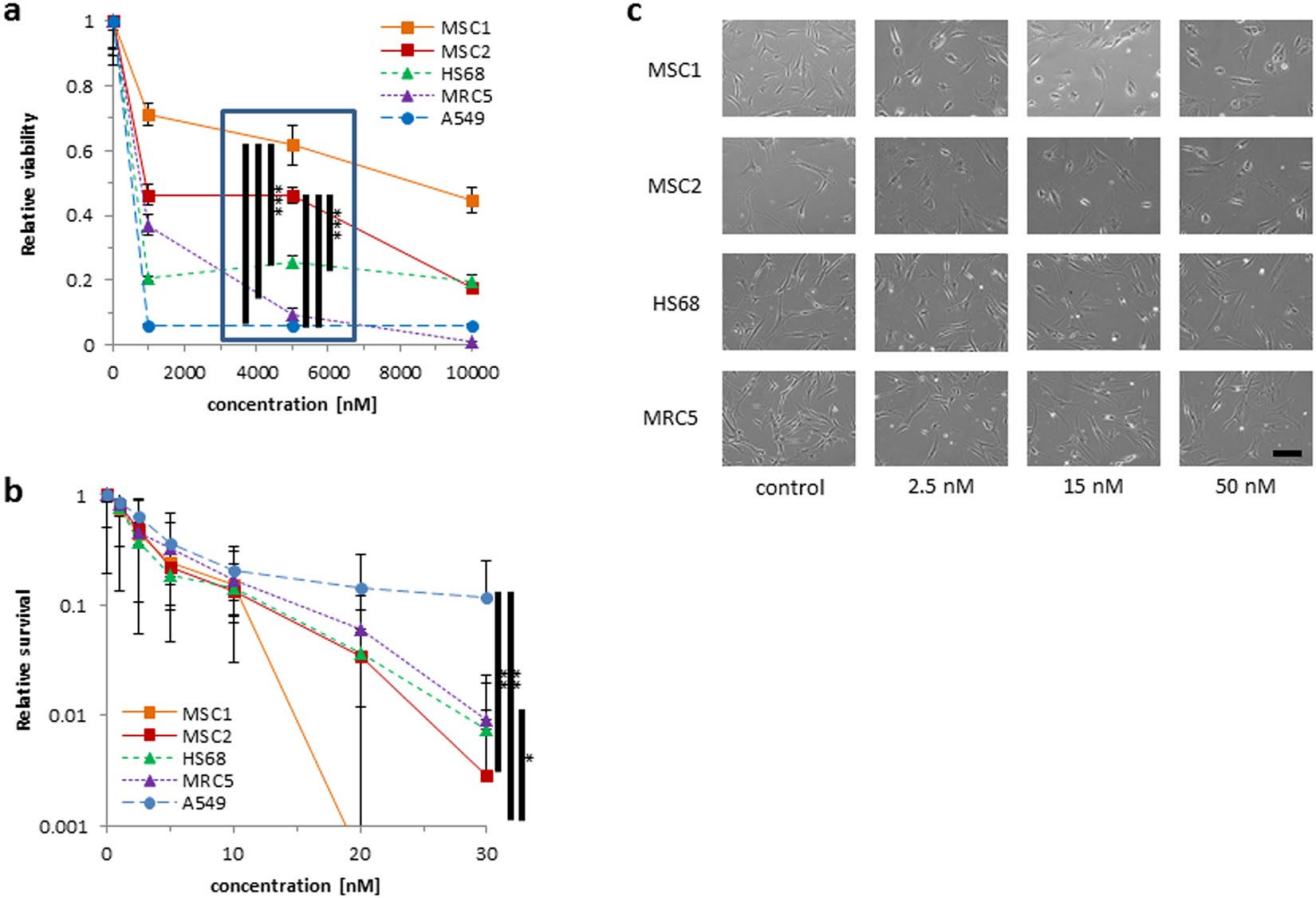
MSCs show stable viability but strongly reduced clonogenic survival after paclitaxel treatment (**a**) MTS assay data showing viability of two different MSCs, two adult fibroblast cell lines and one taxane-responsive lung cancer cell line after treatment with paclitaxel. The blue box represents the clinically relevant peak paclitaxel concentration in patient plasma. (**b**) Clonogenic survival assays for MSCs, fibroblasts and a paclitaxel-responsive lung cancer cell line after paclitaxel treatment. Data are mean +/− SD (n = 3). *P < 0.05; **P < 0.01; ***P < 0.001. (**c**) Representative microscopic images of unstained MSCs and adult fibroblasts with no measurable changes in morphology after treatment increasing concentrations of paclitaxel (20× objective, scale bar 100 µm).

Paclitaxel doses up to 5000 nM resulted in a moderate reduction of viability in both tested MSC samples (Fig. 1a), and MSC survival was significantly superior to that of HS68 (*P* < 0.001 for MSC1 and MSC2) and MRC5 differentiated fibroblasts (*P* < 0.001 for MSC1 and MSC2) and the paclitaxel-responsive A549 lung cancer cell line (*P* < 0.001 for MSC1 and MSC2).

In contrast, the ability for clonogenic survival appeared strongly impeded in both MSC preparations after treatment with low, subclinical doses of paclitaxel and was considerably lower than observed for HS68 (*P* = 0.55 for MSC1, *P* = 0.22 for MSC2), MRC5 (*P* < 0.05 for MSC1, *P* = 0.12 for MSC2) and A549 cells (*P* < 0.01 for MSC1 and MSC2) (Fig. 1b).

### Paclitaxel treatment does not alter MSC morphology or surface marker expression

MSCs and differentiated fibroblasts exhibit a characteristic spindle shape. Treatment with increasing concentrations of paclitaxel did not influence the morphology of the investigated MSC and fibroblast specimens, and no signs of increased apoptosis could be detected by light microscopy at 24 or 48 hours after paclitaxel treatment (Fig. 1c).

**Figure 2:**
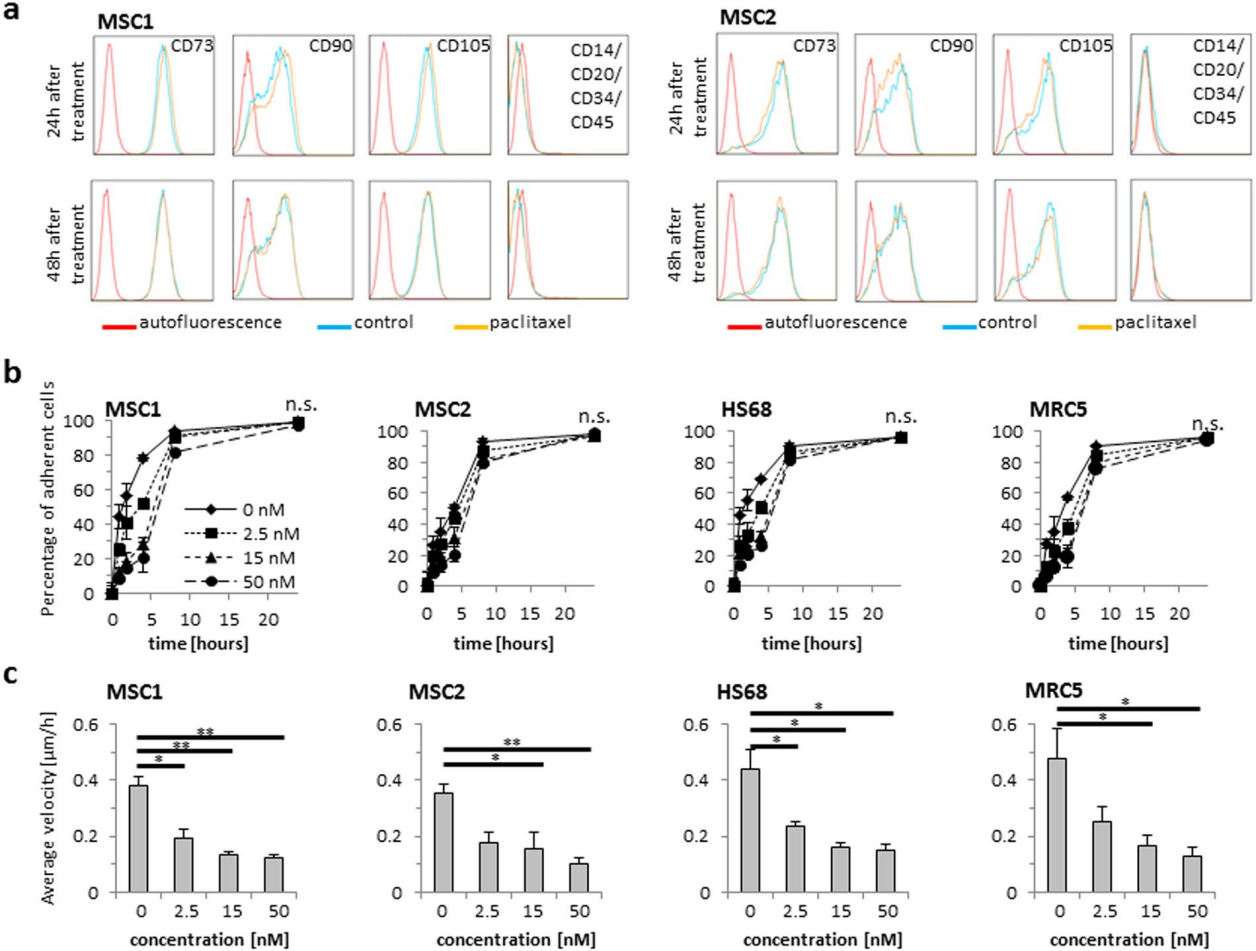
Paclitaxel does not affect the defining surface marker expression, but impairs the adhesion and migration capabilities of MSCs. (**a**) Representative flow cytometry analyses of defining positive MSC markers CD73, CD90 and CD105 and negative markers CD14, CD20, CD34 and CD45 at 24 and 48 hours after treatment with 15 nM paclitaxel. (**b**) Relative adhesion rates of MSCs and differentiated fibroblasts up to 24 hours after treatment with paclitaxel (n = 5). (**c**) Average velocity of MSCs and differentiated fibroblasts after paclitaxel treatment. Data are mean +/− SD. n.s.: not significant; *P < 0.05; **P < 0.01.

Beyond their typical morphology, MSCs are characterized by a unique pattern of surface markers^16^. Expression of established surface markers was measured by FACS analysis at 24 and 48 hours after 24-hour treatment with 15 nM paclitaxel. Levels of positive stem cells markers CD73, CD90 and CD105 were found unaltered at both tested time points, and the absence of the negative markers CD14, CD20, CD34 and CD45 was also not influenced by paclitaxel treatment (Fig. 2a).

### MSCs maintain their adhesion potential but demonstrate reduced migratory velocity after paclitaxel treatment

The potential for adherence to plastic surfaces is a defining characteristic of MSCs; therefore, stem cell adhesion was assessed for up to 24 hours after paclitaxel treatment. In general, MSC adherence was found not reduced after treatment with increasing concentrations of paclitaxel (Fig. 2b). There was a small, dose-dependent delay in adhesion observed both in MSCs and differentiated fibroblast samples; however, after 24 hours, attachment rates did not significantly differ between untreated and paclitaxel-exposed MSCs or fibroblasts (MSC1: *P* = 0.99; *P* = 0.40; *P* = 0.15 for 2.5 nM, 15 nM, 50 nM, respectively; MSC2: *P* = 0.26; *P* = 0.30; *P* = 0.57; HS68: *P* = 0.99; *P* = 0.80; *P* = 0.71; MRC5: *P* = 0.53; *P* = 0.63; *P* = 0.29).

Cellular migration was assessed by time-lapse microscopy over a time period of 24 hours after treatment. Exposure to paclitaxel demonstrated a strong, dose-dependent reduction of average cellular velocity in MSC1 (*P* < 0.05 for 2.5 nM, *P* < 0.01 for 15 and 50 nM) and MSC2 (*P* < 0.05 for 15 nM, *P* < 0.01 for 50 nM) samples (Fig. 2c). Similarly, the migratory potential of differentiated fibroblasts was also found strongly reduced in a dose-dependent manner (*P* < 0.05 for all concentrations in HS68 cells, *P* < 0.05 for 15 and 50 nM in MRC5 cells).

### Paclitaxel treatment causes only minimal effects on the cytoskeletal morphology of MSCs

**Figure 3:**
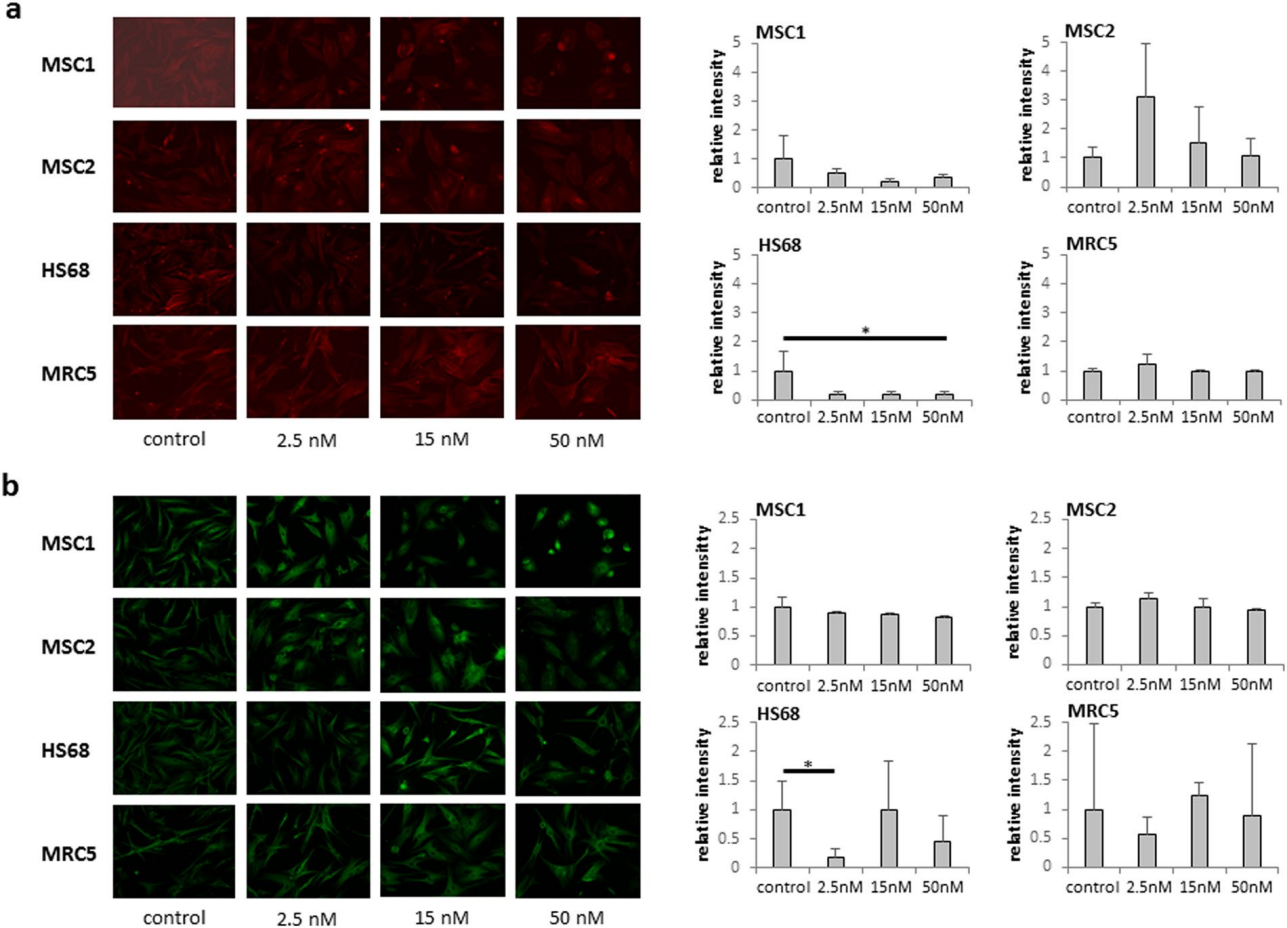
Paclitaxel causes only minimal changes in the cytoskeletal morphology. (**a**) Actin labeling in MSCs and differentiated fibroblasts after different concentrations of paclitaxel. (**b**) Tubulin immunostaining in MSCs and differentiated fibroblasts after paclitaxel treatment. *P < 0.05; **P < 0.01.

Cytoskeletal dynamics have been shown to correlate with the migratory velocity of mesenchymal cells; therefore potential influences of paclitaxel treatment on the cytoskeletal architecture were investigated by fluorescence stainings. In both MSC preparations, exposure to paclitaxel concentrations up to 50 nM had no significant impact on the actin cytoskeleton, and actin signaling was only significantly reduced in HS68 fibroblasts (*P* < 0.05) (Fig. 3a).

In contrast, the tubulin fluorescence signal appeared considerably more heterogenous throughout MSCs and fibroblasts with incipient bundling of mictotubules, while no clear or dose-dependent change in the microtubule staining intensity could be observed for any of the investigated cell types (Fig. 3b). Therefore, no correlation could be detected between the observed reduction in migratory velocity and any alterations in the cytoskeletal architecture for any of the cell lines.

### Paclitaxel impedes the differentiation potential of MSCs

**Figure 4:**
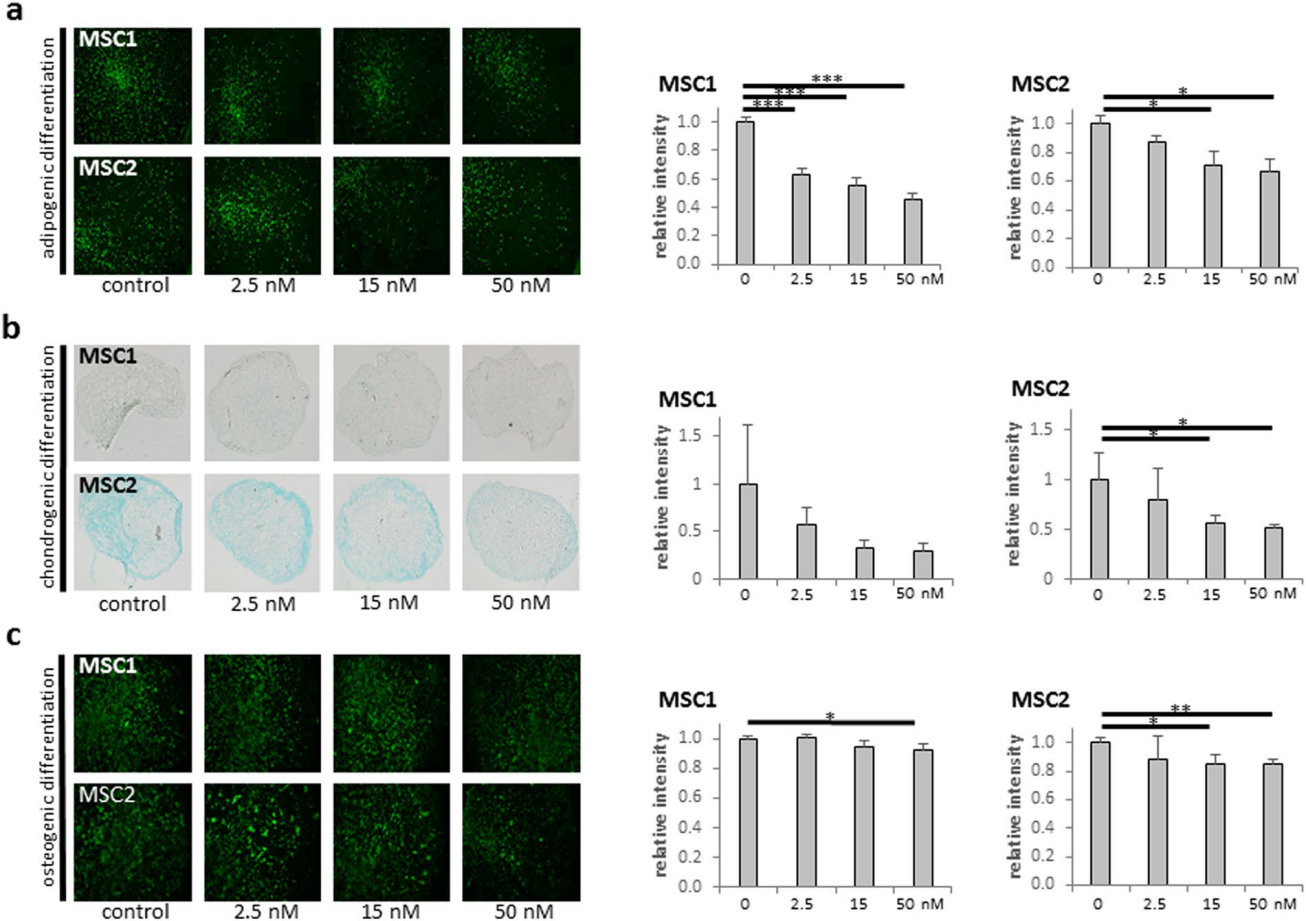
Paclitaxel treatment reduces the multi-lineage differentiation potential of MSCs. (**a**) BODIPY lipid staining of MSC1 and MSC2 samples after treatment with paclitaxel to assess induced adipogenic differentiation. (**b**) Alcian blue staining for induced chondrogenic differentiation of MSCs after exposure to paclitaxel. (**c**) OsteoImage™ staining for induced osteogenic differentiation of MSCs following paclitaxel treatment. Relative staining intensities were measured to quantify differentiation levels after differentiation. Data are mean +/− SD. *P < 0.05; **P < 0.01; ***P < 0.001.

The ability for differentiation along the adipogenic, chondrogenic and osteogenic lineages is a defining hallmark of MSCs^16^. To investigate a potential influence of paclitaxel on the differentiation potential of human MSCs, induced differentiation was quantified by immunocytochemical analyses. A strong, dose-dependent reduction in the ability to undergo induced adipogenic differentiation was observed after paclitaxel treatment both in MSC1 (*P* < 0.001 for all concentrations) and MSC2 (*P* < 0.05 for 15 and 50 nM) (Fig. 4a). Similarly, the chondrogenic differentiation potential of MSCs was reduced by more than half after exposure to increasing concentrations of paclitaxel, although significance was not reached for MSC1 (*P* n.s. for all concentrations in MSC1, *P* < 0.05 for 15 and 50 nM in MSC2) (Fig. 4b). The ability for osteogenic differentiation is commonly preserved in MSCs even after reduction of the adipogenic and chondrogenic differentiation abilities^24,25^. However, paclitaxel treatment resulted in a small but significant dose-dependent reduction in the ability for induced osteogenic differentiation in MSC1 (*P* < 0.05 for 50 nM) and MSC2 (*P* < 0.05 for 15 nM, *P* < 0.01 for 50 nM) stem cell preparations (Fig. 4c).

### Paclitaxel treatment minimally affects apoptosis, but results in strong induction of senescence in MSCs

**Figure 5:**
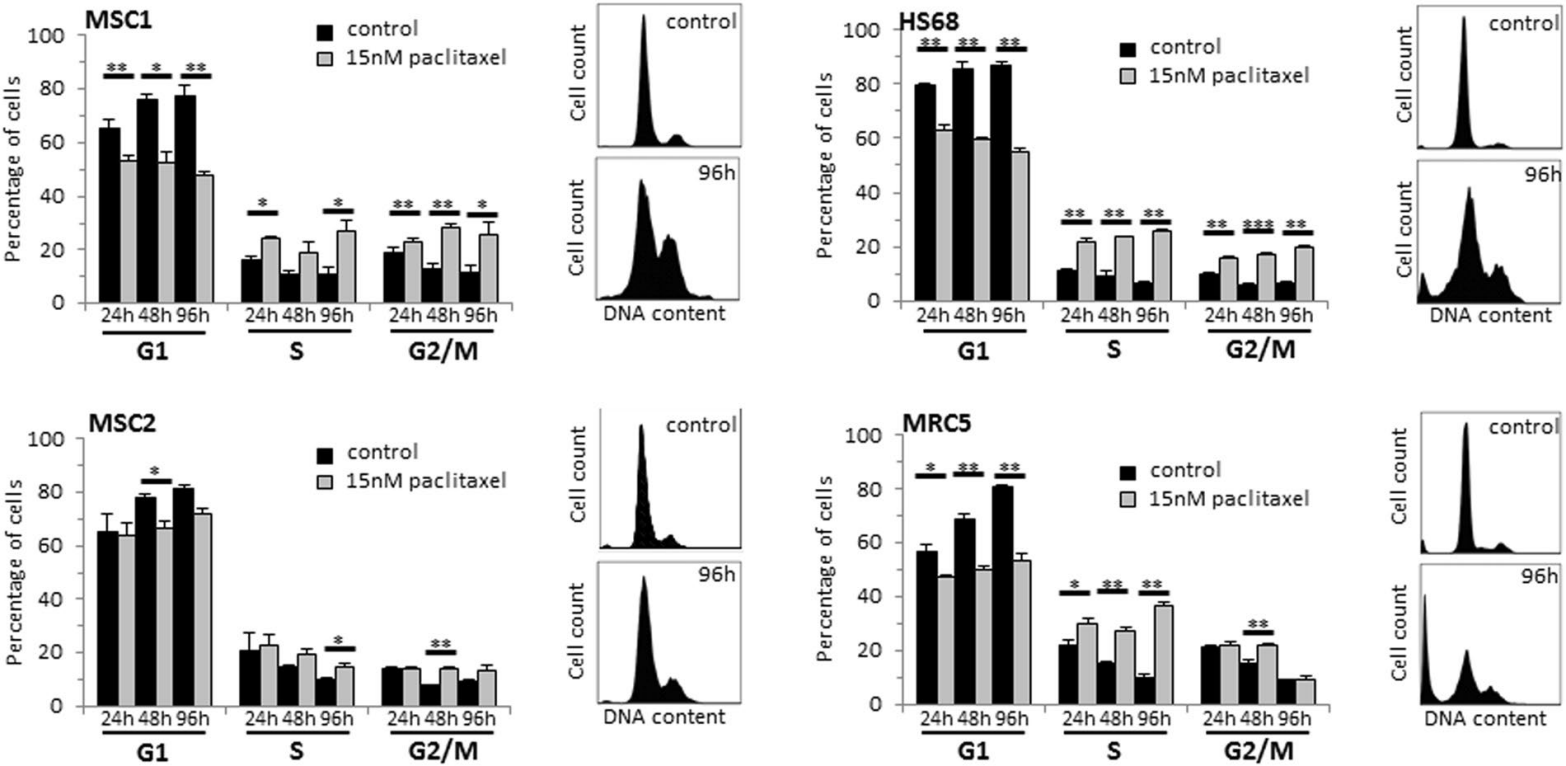
MSCs exhibit a prolonged arrest in G2 phase of the cell cycle after paclitaxel treatment. Cell cycle distribution over time of two MSC preparations and two differentiated fibroblast cell lines after 24-hour treatment with 15 nM paclitaxel. *P < 0.05; **P < 0.01; ***P < 0.001.

The influence of paclitaxel on the cell cycle and induction of apoptosis was measured by flow cytometry. Exposure to 15 nM paclitaxel led to a prolonged accumulation of MSCs in the G2 phase of the cell cycle for up to 96 hours (Fig. 5). HS68 skin fibroblasts exhibited similarly increased levels of G2 phase cells after paclitaxel treatment, whereas MRC5 lung fibroblasts demonstrated only a small transient increase in G2 phase cells at 48 hours.

**Figure 6:**
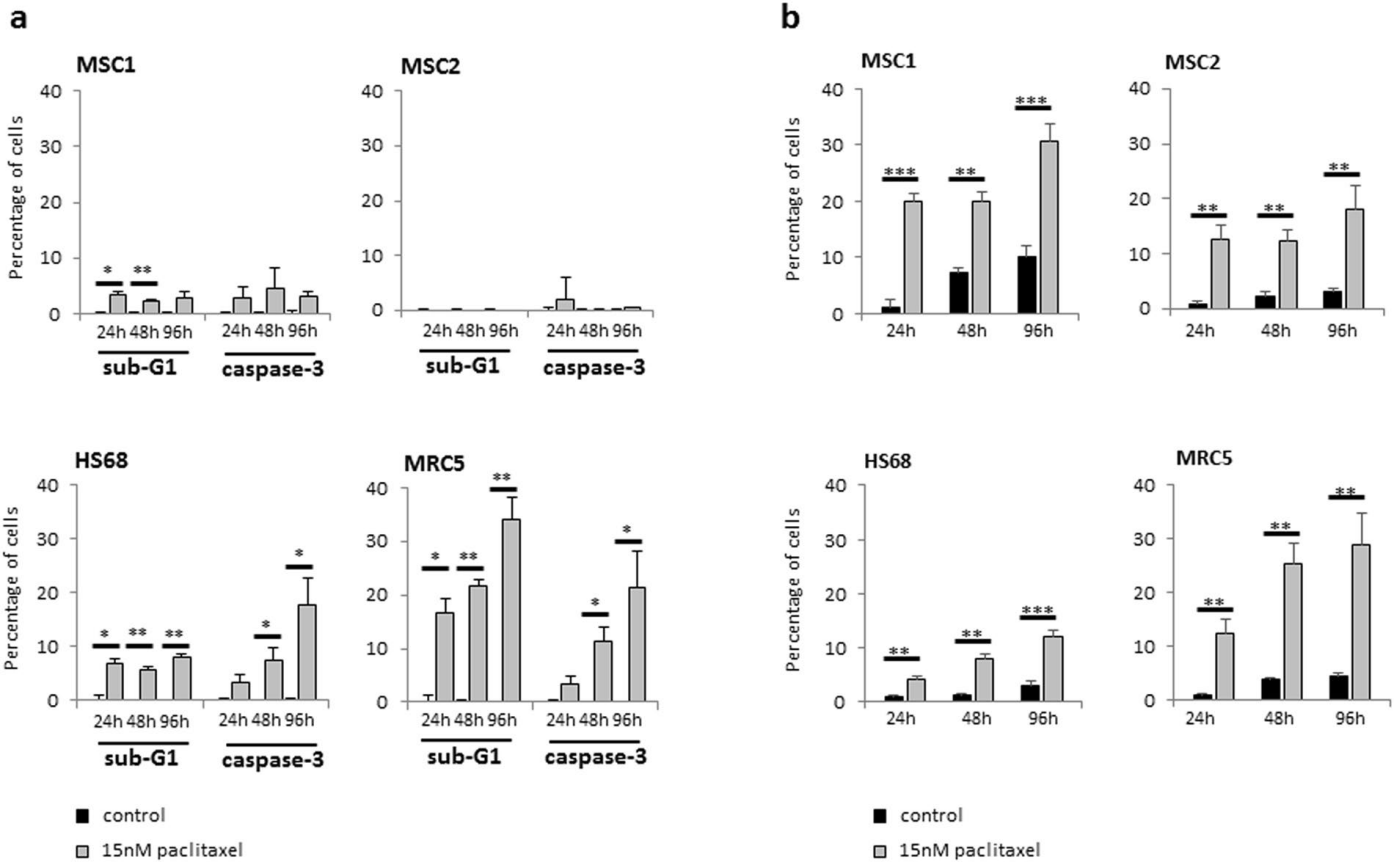
MSCs undergo premature senescence rather than apoptosis after paclitaxel treatment. (**a**) Percentage of apoptotic MSCs and adult fibroblasts above control level at various time points after treatment with 15 nM paclitaxel as assessed by sub-G1 population and caspase-3 activation. (**b**) Percentage of β-GAL-positive cells after treatment with 15 nM paclitaxel. **P < 0.01; ***P < 0.001.

Paclitaxel-mediated induction of apoptosis was assessed by measuring the percentage of cells showing caspase-3 activation and cells with sub-G1 DNA content. MSC samples did not exhibit a general increase in apoptosis after paclitaxel treatment, and the fraction of apoptotic cells remained below 5% for all tested time points (Fig. 6a). In contrast, both tested differentiated fibroblasts revealed a strong paclitaxel-induced increase in apoptosis over time with around 20% of caspase-3-positive cells at 96 hours after treatment (*P* < 0.05 for HS68 and MRC5).

Induction of premature senescence after treatment with paclitaxel was assessed by staining of senescence-associated β-galactosidase (β-GAL). Both tested MSCs exhibited a swift and significant increase in senescent β-GAL-positive cells ranging between 12 and 20% of cells at 24 hours after treatment (*P* < 0.001 for MSC1, *P* < 0.01 for MSC2) and between 20 and 30% after 72 hours (*P* < 0.001 for MSC1, *P* < 0.01 for MSC2) (Fig. 6b). Differentiated fibroblasts demonstrated heterogeneous effects regarding premature senescence with low levels of β-GAL-positive cells ranging between 4 and 12% in HS68 and higher levels between 12 and 30% in MRC5 cells.

## Discussion

MSCs have been previously shown resistance to various anti-cancer treatments such as platinum compounds, topoisomerase inhibitors or ionizing radiation^26–29^. However, systematic analyses regarding the influence of the chemotherapeutic treatments on the functional characteristics of MSCs are needed to investigate the stem cells’ potential use for regeneration of chemotherapy-induced tissue damage. Here, we investigated the influence of taxane treatment on the survival, stem cell traits and functional capabilities of human bone marrow-derived MSCs. We could show that MSC survival and proliferation were strongly affected after exposure to paclitaxel, while the viability of MSCs was largely preserved at treatment doses and exposure times that mimicked the condition of patients undergoing paclitaxel chemotherapy, MSC clonogenic proliferation was massively reduced even at 100-fold lower paclitaxel concentrations^23^. The observed inactivation of the proliferative potential of MSCs even by low concentrations of paclitaxel is in line with previous data suggesting that upon taxane exposure, MSCs adopt a fibroblast-like non-proliferative state^30^. Similarly, cellular proliferation has been reported to decrease in the functionally comparable adipose-derived multipotent mesenchymal stromal cells (adMSCs) after paclitaxel treatment, and rats treated with paclitaxel exhibited lower numbers of proliferative adMSCs after paclitaxel injections^31,32^. Additionally, there is evidence that the inhibition of the stem cells’ proliferative potential is irreversibly damaged after paclitaxel treatment with incomplete recovery within several weeks after taxane withdrawal^31^.

Given the preserved metabolic viability of MSCs coinciding with a strong impairment of proliferation, it is conceivable that paclitaxel induces a quiescent state in these cells, and indeed, we observed a strong induction of premature cellular senescence and suppression of apoptosis induction in paclitaxel-treated MSCs. Similarly, previous publications reported only small increases in apoptosis in MSCs and adMSCs even at considerably higher paclitaxel doses and exposure times^32,33^. As a more general phenomenon, it has been suggested that MSCs can escape apoptotic induction also after exposure to various other anti-cancer agents^27,30,34^. The observation of low apoptosis induction in MSCs has been attributed to an impaired p73-dependent activation of pro-apoptotic proteins, an inhibition of the TRAIL pro-apoptotic pathway and strong constitutive expression of several anti-apoptotic factors like Bcl-2 and Bcl-xL^35–37^. In our dataset, MSCs exhibited strong increases in senescence-associated β-galactosidase stainings even after low doses of paclitaxel; this may explain the observed inactivation of cellular proliferation in taxane-treated MSCs while preserving their metabolic viability. It has been hypothesized that premature senescence is the main coping mechanism by which MSCs avoid proliferation after damage to their cellular functions^38,39^. Regulation of premature senescence in MSCs has been attributed both to key regulatory proteins such as pRB and p53 and to the cyclin-dependent kinase inhibitor 2 A (p16-INK4A), although the exact mechanism and role of premature senescence in MSCs remains to be elucidated^40–42^.

Beyond the paclitaxel-induced reduction of MSC proliferation, we found that paclitaxel significantly impaired the functional characteristics of these stem cells. The ability for cellular movement is a prerequisite for endogenous MSCs to migrate to damaged tissues and participate in regenerative processes^43^. In our dataset, even very low paclitaxel doses led to a significant dose-dependent inhibition of MSC motility. The ability of MSCs to migrate has been linked to the function of actin filaments in MSCs, and MSC actin remodeling has been observed after treatment with other chemotherapeutic agents^27,44,45^. However, in our dataset, no structural changes in the actin cytoskeleton could be observed after paclitaxel treatment. Similarly, MSC adhesion as an established hallmark of these stem cells was delayed in a dose-dependent manner. The ability of MSCs to undergo induced multi-lineage differentiation is another defining function of MSCs. Treatment with low doses of paclitaxel resulted in a profound and dose-dependent reduction of the stem cells’ potential for adipogenic, chondrogenic and osteogenic differentiation. The influence of taxanes on differentiation of human MSCs has been subject to previous investigations as the differentiation potential is a key requirement for these stem cells’ regenerative function. While one publication showed that paclitaxel resulted in severely impaired adipogenic differentiation at doses as low as 10 nM, another report claimed no influence of taxane treatment on the adipogenic and osteogenic differentiation potential, however without presenting the respective data in the publication^30,33^. A link between a reduced differentiation potential of MSCs and the induction of premature senescence has been previously suggested, and accordingly, we observed a strong increase in β-GAL-positive MSCs, coninciding with the partial abrogation of the stem cells’ ability for induced differentiation after paclitaxel treatment^46^.

Further *in-vivo* data may help to corroborate our findings, as the generalizability of the reported observations may be limited by the artificial *in-vitro* MSC model used here. While this model helps to clearly characterize the influence of taxanes on the defining stem cell traits and cellular functions, it does not take into account the potentially relevant influences of the MSCs’ microenvironment and the stem cells’ interaction with other cell types in the bone marrow niche that may also influence cellular taxane sensitivity.

The observed functional impairment of bone marrow-derived MSCs after paclitaxel treatment may be of clinical importance, as the inhibition of the bone marrow function is commonly the dose-limiting toxicity of paclitaxel treatment regimens^47^. MSCs have been suggested as essential mediators of the bone marrow homeostasis, and the retention, proliferation, differentiation and mobilization of bone marrow-derived hematopoietic stem cells has been shown to be dependent on the secretion of various signaling molecules and cytokines by MSCs^29,48–50^. Therefore, the data shown here may help to explain the often severe and extended myelosuppression observed after paclitaxel-based anti-cancer treatment. Additionally, novel approaches that spare or restore the bone marrow’s functional MSCs after paclitaxel therapy, e.g. by harvesting the stem cells beforehand and re-transplanting them during or after chemotherapy may help to attenuate or avoid severe paclitaxel-induced myelosuppression. However, further *in-vivo* studies are needed to devise and investigate potential MSC-based strategies in order to target bone marrow effects of paclitaxel.

Taken together, our data revealed the taxane-sensitive phenotype of human bone marrow-derived MSCs and showed the impeding influence of taxanes on the defining functional properties of these stem cells. Inhibition of bone marrow-resident MSCs may help to explain the severe bone marrow toxicities commonly caused by taxane-based anti-cancer treatments.

## Methods

### Cells and culture

Human MSC1 and MSC2 mesenchymal stem cell preparations were harvested after written informed consent from the bone marrow of healthy volunteers and isolated as published previously^51,52^. MSCs were cultured in Mesenchymal Stem Cell Growth Medium (Lonza, Basel, Switzerland) with added MSCGM™ Single Quots (Lonza) at 37 °C and 5% CO_2_. HS68 human dermal fibroblasts were purchased from the ATCC (Manassas, USA) and were grown in Dulbecco’s Modified Eagle Medium (Biochrom, Berlin, Germany) with 10% fetal bovine serum and 3.5 g/L glucose. Human MRC5 pulmonary fibroblasts were obtained from the ATCC and were proliferated in Eagle’s Minimum Essential Medium (Sigma-Aldrich, Munich, Germany) supplemented with 10% fetal bovine serum. A549 lung carcinoma cells were received from the ATCC and grown in Roswell Park Memorial Institute-1640 medium (Lonza) including 10% fetal bovine serum. This study was approved by the independent ethics board of the University of Heidelberg (S-348/2004), and all experiments were performed according to the approved guidelines.

### Drug preparation

Paclitaxel stock solution at a concentration of 7 mM was received from the Heidelberg University Hospital central pharmacy and was stored in the refrigerator for up to 7 days. Immediately prior to each experiment, the drug was diluted in culturing medium to the required concentrations. All experimental setups containing paclitaxel were protected from light.

### Viability assays

Cellular viability after drug treatment was measured by the MTS assay. 2 000 cells were plated in each well of a 96-well plate with the addition of 200 µL of culturing medium, and paclitaxel was added in each well to the required concentration. Treated cells were then maintained for further 5 days. 20 µL of 1.9 mg/mL MTS reagent (Promega, Madison, USA) was pipetted in each well and incubated for 2 hours before absorbance measurements at 490 nm were performed on a microplate reader (Tecan, Crailsheim, Germany).

### Clonogenic survival assays

After plating, cells were allowed to attach for 6 hours prior to taxane treatment. Paclitaxel was then added to the cells for 24 hours before medium was replaced and cells were further incubated for 14 days to allow colony formation. Colonies were fixed with 25% acetic acid (v/v) in methanol and then stained with crystal violet solution. Colonies comprising more than 50 cells were counted on a light microscope. All clonogenic assays were performed in triplicate. The surviving fraction of cells was calculated by the formula (#colonies/#plated cells)_treated_/(#colonies/#plated cells)_untreated_.

### Surface marker expression

MSCs were grown to 80% confluency and then treated with paclitaxel at a concentration of 15 nM for 24 hours. Cells were then harvested at 24 and 48 hours, and the expression of positive and negative surface markers was examined using the MSC Phenotyping Kit (Miltenyi Biotec, Bergisch-Galdbach, Germany) based on the proposed minimal defining criteria for MSCs^16^. The staining was carried out according to the manufacturer’s instructions, and stained samples were assessed on a FACSCanto flow cytometer (Becton-Dickinson, Heidelberg, Germany). Extracted data were analyzed using FlowJo 7.6.5 software (FlowJo LLC, Ashland, USA).

### Adhesion measurements

Cells were exposed to paclitaxel for 24 hours at concentrations ranging between 2.5 and 50 nM. 100 cells were then transferred to each well of a 96-well plate, and attached cells over time were counted on a light microscope. The attachment efficiency was assessed as the ratio between attached and plated cells. All measurements were performed at least in triplicate.

### Migration measurements

Cells were grown to a confluence of 30–50% and treated with different concentrations of paclitaxel indicated in the Results section for 24 hours. Cell movement was observed and images taken every 7 minutes over a time period of 35 hours by time-lapse microscopy using an IX70 inverted microscope equipped with an incubator box (Olympus, Hamburg, Germany). Cell movement tracks were outlined and measured in length every 7 minutes over the observation period. Individual track lengths were quantified using ImageJ software (National Institutes of Health, Bethesda, USA), and the average velocity was calculated as the average track length per time. At least 10 cells from three different locations of each well were measured for each experimental condition.

### Cytoskeletal stainings

The cytoskeletal architecture of paclitaxel-treated cells was assessed by fluorescence stainings. Cells were fixed with 4% paraformaldehyde and permeabilized with 0.2% Triton X-100 for 5 min, and unspecific antibody binding was blocked by incubation with 5% normal goat serum for 1 hour. Actin filaments were stained with 100 nM Alexa Fluor-633 phalloidin (Life Technologies, Darmstadt, Germany) in PBS for 30 min. Microtubules were visualized by immunostaining using a mouse monoclonal anti-α tubulin antibody (Sigma, Munich, Germany) and a secondary DyLight-488-coupled anti-mouse antibody (Abcam, Cambridge, UK) for 1 hour at room temperature. Nuclei were counterstained with 1 µM DAPI for 5 min. For quantification, at least five images were acquired for each treatment condition with a Keyence BioRevo9000 microscope (Keyence, Neu-Isenburg, Germany) using a 20× objective. Data analysis was performed with ImageJ software (National Institutes of Health, Bethesda, USA).

### Cellular differentiation experiments

To measure the potential of MSCs for induced differentiation, cells were plated in 24-well plates and treated with 2.5 nM, 15 nM or 50 nM paclitaxel for 24 hours, before cells were incubated in regular medium for a further 24 hours. Medium was then replaced by differentiation media, and cells were grown for 21 days. All differentiation media were exchanged twice per week. Adipogenic differentiation was induced by DMEM containing 10% fetal bovine serum, 2 mM L-glutamine, 1 µM dexamethasone, 500 µM 1-methyl-3-isobutylxanthine, 10 µg/mL insulin and 100 U/mL penicillin/streptomycin. Adipogenic differentiation was quantified after cellular staining with 1 µg/mL BODIPY (493/503) (Life Technologies, Darmstadt, Germany) for 20 min. Nuclei were counterstained with 1 µM DAPI for 5 min.

Chondrogenic differentiation was performed using the STEMPRO® Chondrogenesis Differentiation Kit (Gibco Life Technologies, Frankfurt, Germany) according to the manufacturer’s protocol. Differentiated spheroids were fix with 4% paraformaldehyde before freezing at −20 °C and sectioning on a cryomicrotome. Sections were stained in 1% Alcian Blue/3% acetic acid solution for 30 min at room temperature before washing steps using 0.1 M hydrochloric acid, PBS and deionized water.

Osteogenic differentiation medium consisted of DMEM containing 10% fetal bovine serum, 10 mM b-glycerophosphate, 1 µM dexamethasone and 0.2 mM ascorbic acid. After 21 days, differentiated cells were stained with OsteoImage™ Staining Reagent (Lonza, Cologne, Germany), nuclei were counterstained with 1 µM DAPI for 5 min. To quantify adipogenic and osteogenic and differentiation, fluorescence images of whole cells were taken under identical conditions for all experiments on a Keyence BioRevo9000 microscope. Microscopy of Alcian blue-labeled chondrogenically differentiated spheroids was performed on a light microscope. Mean staining intensities were quantified using ImageJ software.

### Cell cycle analyses

To analyze cell cycle effects, cells were treated with 15 nM paclitaxel for 24 hours before harvesting and fixation in ice-cold 70% ethanol. After washing and centrifugation, cells were treated with 1 µg/mL 4′,6-diamidin-2-phenylindol (DAPI) solution containing 200 µg/mL RNase A. Flow cytometry analyses were then performed using a LSR II system (Becton-Dickinson). 10 000 events were counted for each treatment condition, and modeling of cell cycle profiles was performed using FlowJo 7.6.5 software.

### Apoptosis measurements

Cells were mock-treated or treated with 15 nM paclitaxel for 24 hours prior to proliferation for up to 96 hours. Cells were then fixed in 4% paraformaldehyde solution, resuspended in ice-cold 70% ethanol and washed in PBS containing 200 µg/mL RNaseA and 5 g/L bovine serum albumin. After a centrifugation step, a fluorescence-coupled antibody against activated caspase-3 (1:20, BD Pharmingen, Heidelberg, Germany) was added for 1 hour at room temperature. Flow cytometry was performed on a LSR II system, and 10 000 events were counted for each experimental condition.

### Senescence measurements

Cells were grown in 6-well plates and treated with 15 nM paclitaxel for 24 hours prior to proliferation for up to 96 hours. Senescence induction was assessed using a β-galactosidase staining kit according to the manufacturer’s instructions (Cell Signaling Technologies). In summary, cells were washed and fixed before staining with β-galactosidase staining solution at 37 °C overnight. Images of labeled cells were taken on a Keyence BioRevo9000 microscope at 20× magnification, and quantification of staining intensity was performed using ImageJ software.

### Statistical analyses

For data quantification, mean values and standard deviation were calculated from at least three experimental replicates. Statistical analyses were performed using the unpaired Student’s t-test, and two-sided *P*-values < 0.05 were considered significant.

### Data availability

The datasets generated during and/or analyzed during the current study are available from the corresponding author on reasonable request.

## Electronic supplementary material

Supplementary Dataset 1
