# Supplementary material for: Human mesenchymal stem cells lose their functional properties after paclitaxel treatment
Source: Sci Rep. 2018 Jan 10;8:312. doi: 10.1038/s41598-017-18862-1 (PMC5762916; doi:10.1038/s41598-017-18862-1)
Supplement: Supplementary file 1 — Supplementary Dataset 1 [file 41598_2017_18862_MOESM1_ESM.pdf]

Supplementary data

Human mesenchymal stem cells lose their functional properties after paclitaxel treatment

Franziska Münz, Ramon Lopez Perez, Thuy Trinh, Sonevisay Sisombath, Klaus-Josef Weber, Patrick Wuchter, Jürgen Debus, Rainer Saffrich, Peter E. Huber, Nils H. Nicolay

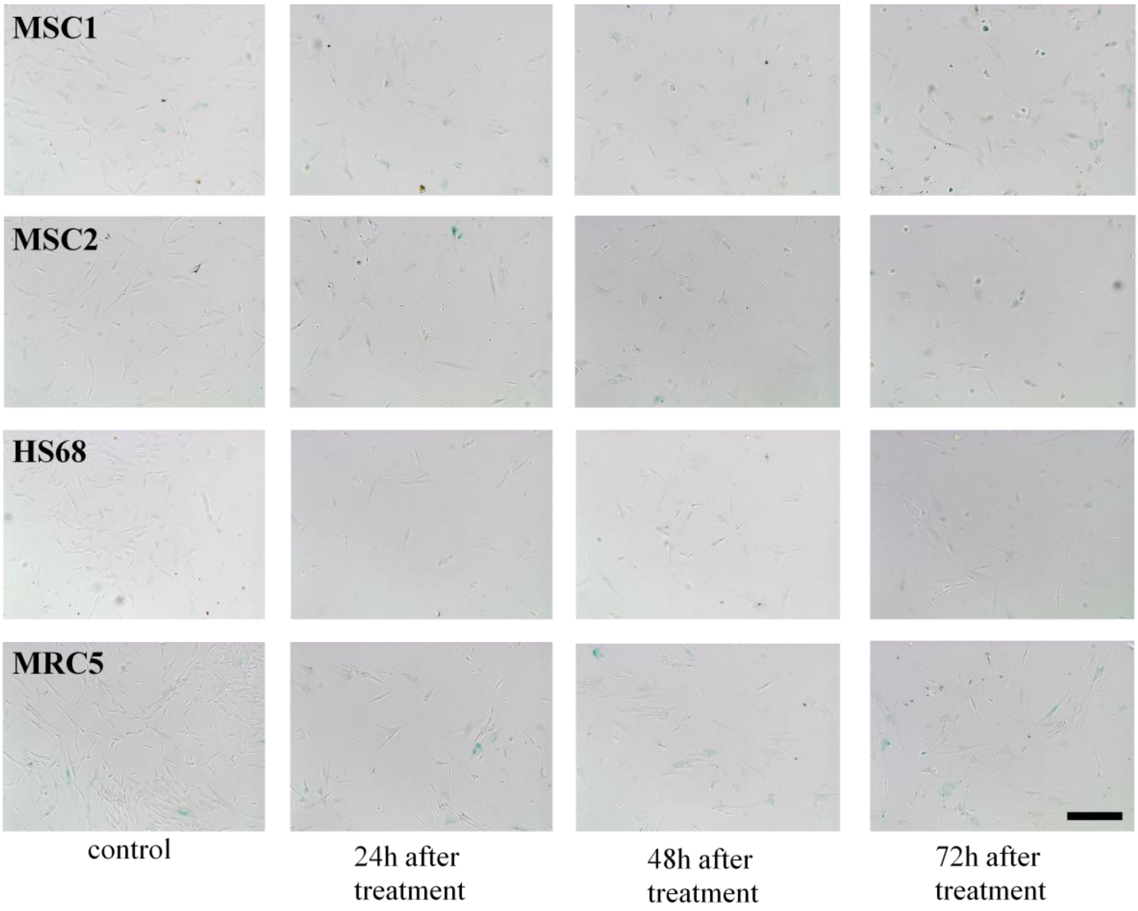

Supplementary figure 1. Representative images of senescence-associated β-galactosidase-stained MSCs and fibroblasts (20x objective, scale bar 100µm).
